# Comparison of Topical Nifedipine With Oral Nifedipine for Treatment of Anal Fissure: A Randomized Controlled Trial

**DOI:** 10.5812/ircmj.13592

**Published:** 2014-08-05

**Authors:** Farzaneh Golfam, Parisa Golfam, Babak Golfam, Puyan Pahlevani

**Affiliations:** 1 Department of Surgery, Mostafa Khomeini Hospital, Shahed University, Tehran, IR Iran; 2 Department of Anesthesiology, Imam Reza Hospital, Kermanshah University of Medical Sciences, Kermanshah, IR Iran; 3 Department of Anesthesiology, Shahid Beheshti University of Medical Sciences, Tehran, IR Iran; 4 Hamadan University of Medical Sciences, Hamadan, IR Iran

**Keywords:** Fissure in Ano, Nifedipine, Sphincterotomy, Endoscopic

## Abstract

**Background:**

Medical sphincterotomy has gained popularity as a treatment for anal fissure. Calcium channel blockers in topical forms could also be appropriate with low adverse effects.

**Objectives:**

This was a prospective randomized controlled trial to compare topical and oral nifedipine in the treatment of chronic anal fissure.

**Patients and Methods:**

A prospective randomized controlled trial was conducted at two centers of Shahed University. One hundred and thirty patients with chronic anal fissure aged 18 to 60 years managed in our clinics were included in this study. The patients were randomly divided into two groups. Sixty-five patients received topical nifedipine (TN) and the same number received oral nifedipine (ON).

**Results:**

Ulcer healing occurred in 43 (73.33%) of topical nifedipine group compared to 29 (49.5%) patients in oral nifedipine, which was significantly different (P < 0.05). Side effects such as headache and flushing in oral nifedipine group were more prevalent than topical nifedipine, which was statistically different. Recurrence rates were the same after six months of follow-up.

**Conclusions:**

Although oral nifedipine can reduce symptom and signs of anal fissure, topical nifedipine has a superior role for anal fissure treatment with higher healing rate and lower side effects.

## 1. Background

Anal fissure is one of the most common diseases in general surgery or proctology clinics. It is a longitudinal tear or split in anoderm just distal to the dentate line often associated with tearing pain with or without rectal bleeding during and after defecation. A minority complain of swelling, pruritus and discharge. Lesions are often in the posterior midline. Skin tags or piles may remain indicating chronic inflammatory changes. The Pathogenesis of anal fissure is uncertain but an accepted theory argues that elevated resting sphincter pressure or muscle spasm leads to relative ischemia especially in posterior midline ulceration, which results in persistence of internal sphincter hypertonia followed by hard stool and higher anal pressure even at rest (1, 2). Most acute anal fissures would heal spontaneously or with conservative therapy. Nonhealing lesions need further medical or surgical treatment. Although with lateral internal sphincterotomy symptomatic improvement and healing are superior to medical therapy, postoperative incontinence rates are noticeable incidence and durability (3). Therefore, alternative modalities need to be sought. "Chemical sphincterotomy" with various compounds has been suggested (4). This alternative option is potentially cost-effective for the management of chronic anal fissure (5). One of the earliest methods of nonsurgical management was botulinum neurotoxin injection (6). Topical medications proposed in the treatment of anal fissure include NO donor ointments such as Isosorbide dinitrate, glyceryl trinitrate, calcium channel blockers, bethanechol, indoramin and potassium channel opener minoxidil. Most previous researches concerned the efficacy of topical agents in anal fissure management. Efficacy of topical diltiazem and nifedipine were offered in our previous studies for the treatment of chronic anal fissure (7, 8). Usage of oral nifedipine was suggested in some studies as a systemic agent effective in anal fissure therapy (8), but no trial was found comparing the two forms of nifedipine. Therefore, this trial was conducted to compare topical nifedipine with oral nifedipine in chronic anal fissure healing, complications and recurrence rate after six months.

## 2. Objectives

This study designed to compare topical and oral nifedipine in the treatment of chronic anal fissure. A prospective randomized controlled trial was conducted. 

## 3. Patients and Methods

A prospective randomized controlled trial was conducted at two centers of Shahed University in Tehran. The study protocol was approved by the ethics committee of our centers and performed in accordance with the principle of the Helsinki declaration and then registered in Iranian Registry of Clinical Trial (IRCT201104166200n1). Study method was explained to all patients and they were informed regarding the efficacy and adverse effects of the two forms of drug. Informed consent was obtained from all patients. In total, 130 patients with chronic anal fissure aged 18 to 60 years managed in our clinics between February 2002 and February 2007 were included in the study. Chronic fissure was defined as midline posterior or anterior fibrotic ulcer with hypertrophic anal papilla and sentinel pile. None of the patients had a history of sexually transmitted disease, TB, IBD, or medically related conditions such as migraine, cardiovascular and other chronic diseases, anorectal surgery or another anorectal disease. Pregnant patients were also excluded. The patients were randomly divided into two groups. Sixty-five patients received topical nifedipine (TN) and the same number received oral nifedipine (ON). In the first group, patients were asked to apply 1 of nifedipine cream (0.5%) to the anal margin and patients in the second group took 10 mg anal nifedipine in three divided doses for four weeks. All patients were advised to increase their intake of fibers and usage of sitz bath for 10-15 minutes, 2-3 times daily. Eight patients (two patients of TN group and six patients of ON group) who had continuous severe symptoms after two weeks were excluded from the study. Three patients of TN did not continue their medical management. Therefore, the ultimate number of patients included in the study reduced to 119 (60 patients in TN and 59 patients in ON group). A visual analogue scale was devised between 0 and 10. Patients were asked to give 0 point to no pain and 10 for the worst pain they ever experienced. On the first visit and regular follow-up visits (1w, 2w, 4w), the position of fissure, pain score, BP, PR, headache and other complications were recorded. Complete healing was defined with complete epithelialization of ulcer bed at visual assessment and no pain. After complete healing, the patients were followed at 2, and 6 months or earlier if symptoms had relapsed. Statistical analysis was conducted using SPSS for windows ver.10. We checked normal distribution of variables. Students T-test was used for quantitative data and chi-square and U-Mann-Whitney for qualitative data. Quantitative data were presented as mean, standard deviation and range. P < 0.05 was considered significant.

## 4. Results

The TN group included 60 patients, 36 female (60%) and 24 male (40%). Their mean age was 33.71 ranging from 18 to 60 years. The ON group included 59 patients, 32 female (54.23%) and 27 male (45.77%). Their mean age was 32.61 from 20 to 55 years. No significant difference was detected between gender and age distributions of patients as shown in Table 1.

**Table 1.** Comparison of Gender and Age ^a,b^

Groups	Female	Male	Mean Age
**TN**	36 (60)	24 (40)	33.71 ± 6.31
**ON**	32 (54.23)	27 (45.77)	32.61 ± 6.03

^a^ Abbreviations; TN, topical nifedipine; ON, oral nifedipine

^b^ Data are presented as Mean ± SD or No. (%).

The fissures position were posterior in 84 (70.54%) patients, anterior in 17 (14.28%) patients and both anterior and posterior in 18 (15.12%) patients. In both groups, pain score ranged from 5 to 8 in visual analogue scoring (mean VAS (Visual Analogue Scale) was 5.8 in TN group and 5.07 in ON group).

**Table 2.** Position of Anal Fissure and Mean VAS Score^a^

Groups	Anterior	Posterior	Anterior and Posterior	Mean VAS
**TN**	10	42	8	5.8
**ON**	7	42	10	5.24
**Total, No. (%)**	17 (14.58)	84 (70.59)	18 (15.12)	

^a^ Abbreviations: TN; topical nifedipine, ON; oral nifedipine, VAS; visual analogue scale.

Preintervention VAS scores in the two groups were comparable (P = 0.08). In TN group, after four weeks treatment, the fissure healed in 42 (70%) patients assessed with examination and patients reported no pain or other symptoms. Reduction of pain (mean VAS: from 5.8 to 2.1) in 15 patients was observed. Three patients who reported no pain had not complete healing in examination. Two patients with persistent ulcer after two weeks additional course of TN (in the same 6w treatment) were healed. In ON group 30 (50.84%) patients had 0 score in VAS after four weeks of treatment and in examination 24 patients (49.15%) were healed. Additional course (2w) for one patient in ON group who had no pain with permanent ulcer had no effect on ulcer healing. Therefore, ultimate ulcer healing after six weeks in the two groups were 43 (73.33%) patients in TN and 29 (49.15%) patients in ON group, which was significantly different (P = 0.033). In TN group there were no significant adverse effects, there were only mild headache in four patients (6.6%) who were treated with acetaminophen. BP parameters had not significant change in the two groups at every follow-up visit (P = 0.09). There were only one episode of orthostatic hypotension in ON group and mild headache in five patients of this group. No change in PR was noted. A significant number of ON group (25 patients) experienced flushing. Four of healed patients in ON group had recurrence within the first two months of follow-up. Therefore, the recurrence rates were the same in the two groups. In six months follow-up, recurrence in TN and ON groups were four (6.6%) and three (5.8%) patients, respectively.

## 5. Discussion

The current evidence shows that anal fissure is due to anal sphincter hypertonicity and secondary local ischemia, especially in posterior commissure (9, 10). Some studies demonstrated low perfusion in posterior commissure as common site of anal ulcer with post mortem angiographies (11) and Doppler laser flowmetry investigation (12). On the other hand, within pressure accompanied by rise in blood supply and fissure treatment is noted after medical or surgical sphincterotomy (13-15). As we know cytoplasmic calcium is important for smooth muscle contraction and after Coccia et al. used nifedipine in the treatment of achalasia (15), Calcium channel blockers were evaluated for anal fissure treatment. Indeed some researches recommended that chronic anal fissure could be treated with Ca-channel blockers (Level evidence: class I, Grade of recommendation: A) (16).

Antropoli et al. reported reduction in maximum resting pressure in most patients who received 0.2% nifedipine ointment (17). Perrotti et al. in a randomized double blind study reported similar effect for topical nifedipine (18). For assessment of oral nifedipine effect on chronic anal fissure, three manometric studies were conducted reporting reduction in maximum resting pressure in patients who received oral nifedipine (19-21). In our study, topical nifedipine had a clinically and statistically significant success rate for pain relief and ulcer healing at six weeks compared to oral nifedipine. Topical nifedipine caused minimal side effects as compared to oral nifedipine. Considering the limited number of sample group, we concluded that oral nifedipine caused side effects such as flushing and headache, while topically treated group showed minimal side effects. It seems that topical nifedipine brings about lower side effects and is a more effective treatment compared to its oral form.
